# Culturally transmitted song exchange between humpback whales (*Megaptera novaeangliae*) in the southeast Atlantic and southwest Indian Ocean basins

**DOI:** 10.1098/rsos.172305

**Published:** 2018-11-28

**Authors:** Melinda L. Rekdahl, Ellen C. Garland, Gabriella A. Carvajal, Carissa D. King, Tim Collins, Yvette Razafindrakoto, Howard Rosenbaum

**Affiliations:** 1 Wildlife Conservation Society, Ocean Giants Program, Global Conservation Program, 2300 Southern Boulevard, Bronx, NY 10460-1099, USA; 2 School of Biology, University of St Andrews, St Andrews, Fife KY16 9TH, UK; 3 School of Marine and Atmospheric Sciences, Stony Brook University, Stony Brook, NY 11794-5000, USA; 4 COSAP Sahamalaza Miaro Dugong C/O Madagascar National Parks Sahamalaza, Analalava, Maromandia, Madagascar

**Keywords:** song, humpback whale, population structure, cultural transmission, Africa

## Abstract

In migratory marine species, investigating population connectivity and structure can be challenging given barriers to dispersal are less evident and multiple factors may influence individual movement patterns. Male humpback whales sing a song display that can provide insights into contemporary connectivity patterns, as there can be a cultural exchange of a single, population-wide shared song type with neighbouring populations in acoustic contact. Here, we investigated song exchange between populations located on the east and west coasts of Africa using 5 years of concurrent data (2001–2005). Songs were qualitatively and quantitatively transcribed by measuring acoustic features of all song units and then compared using both Dice’s similarity index and the Levenshtein distance similarity index (LSI) to quantitatively calculate song similarity. Song similarity varied among individuals and potentially between populations depending on the year (Dice: 36–100%, LSI: 21–100%), suggesting varying levels of population connectivity and/or interchange among years. The high degree of song sharing indicated in this study further supports genetic studies that demonstrate interchange between these two populations and reinforces the emerging picture of broad-scale connectivity in Southern Hemisphere populations. Further research incorporating additional populations and years would be invaluable for better understanding of fine-scale, song interchange patterns between Southern Hemisphere male humpback whales.

## 1. Introduction

Population structure and connectivity among populations are important factors to consider in conservation and management decisions [1–4]. Understanding population connectivity and structure can be particularly challenging for migratory marine species as barriers to dispersal are less evident than terrestrial ecosystems, and marine populations tend to be more homogeneous [5,6]. A number of factors may influence population structures in migratory marine species, including ecological, environmental, genetic and behavioural processes, which may operate over historical or contemporary time scales [7]. On contemporary time scales, for example, individual movement patterns and population connectivity can change rapidly in response to changing oceanographic conditions or foraging opportunities, which can become more pronounced under a changing climate regime [8,9]. While the fields of genetics and more recently genomics [10] have proved invaluable for understanding population structure and connectivity of marine mammals over historical time scales, limitations exist for interpreting contemporary movement and connectivity patterns using these methods. Using additional methods that can contribute behavioural and/or environmental information at a higher temporal resolution can prove beneficial for informing conservation and management decisions [11].

Humpback whales (*Megaptera novaeangliae*) are one of the most well-studied migratory marine mammal species. In the Southern Hemisphere, migratory routes are complex and discrete populations may have opportunities for acoustic contact and population mixing at certain points during migration or on feeding grounds. Humpback whale population structure and connectivity have typically been investigated using genetic and photo-identification (photo ID) studies. Mitochondrial DNA (mtDNA) and nuclear DNA (e.g. microsatellite) studies have investigated gene flow [12–14], while photo ID studies using mark–recapture techniques have been undertaken to assess population interchange. However, genetic and photo ID studies generally require a high level of effort to collect relatively small sample sizes. Also, these methods have limitations for understanding contemporary movement patterns, as genetic data typically operate over longer temporal scales and are generally costly to collect. Acoustic data for this species can provide another complementary and cost-effective means for assessing population structure and connectivity through the analysis of annual changes in the male song breeding display [15].

Humpback whale songs are complex and composed of sequenced vocalizations typically sung in a specific pattern [16]. It is generally agreed that songs continually evolve within and between years [17–19]; however, all males within a population typically maintain the same song at any point in time [16,18,20,21]. Garland *et al*. [22] demonstrated that song may be learned in segments from conspecifics (e.g. ‘horizontal cultural transmission’) [23,24]. Presumably, male humpback whales must be close enough to hear singers for song learning to occur. There can be considerable intra- and inter-individual variability in song composition within populations, which may be due to the behavioural or physiological mechanisms that are not currently well understood. Nonetheless, the degree of variability in an individual's songs does not negate the population-wide conformity by males to a particular song type (version of the song) at any point in time, and intra-population variability is generally less than inter-population variability in the song [15]. As such, at any time during a breeding season, songs can be used to distinguish between male singers in different populations (as a proxy for wider population-level differences) based on differences in the composition of their songs [15]. Song exchange between distinct populations does occur and the amount of song similarity between populations appears to be dependent on geographical proximity, with populations within the same ocean basin tending to sing more similar songs than those between ocean basins due to more opportunity for acoustic acquisition [25–31]. Recently, songs have been used to investigate connectivity between western and central South Pacific Ocean humpback populations [15], a result that supported and refined the current understanding of population structure inferred from genetics and photo ID data.

**Figure 1.**
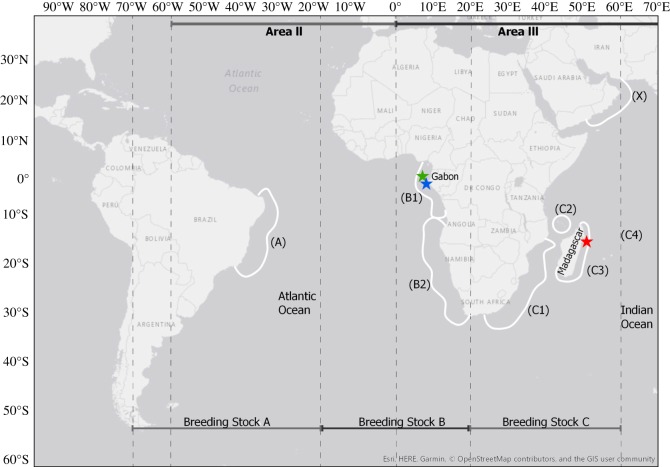
Map adapted from Rosenbaum *et al.* [12] that illustrates sub-stock structure of breeding stocks B and C in relation to the IWC's Southern Ocean feeding ground management areas. Humpback whale songs were recorded at three study sites: Iguela (green star), Mayumba (blue star) and Antongil Bay (red star). Samples from Iguela (green) and Mayumba (blue) were presumably collected from individuals in the breeding sub-stock B1 (BSB1), but with uncertainty regarding sub-stock boundaries we used the population-level demarcation (BSB) when referring to the population. Samples representing breeding stock C (BSC) were sampled from sub-stock C3 (BSC3) in Antongil Bay (red), Madagascar.

In the Southern Hemisphere, the International Whaling Commission (IWC) currently recognizes seven distinct breeding stocks of humpback whales (BSA–BSG). Although breeding stocks are considered to be genetically distinct, they have varying degrees of population structure and a number of populations have been divided into sub-stocks [12–14]. The IWC divided the west African population in the southeast Atlantic (BSB) into two sub-stocks (1 and 2) [12,13,32] and the east African population (BSC) in the southwest Indian Ocean into four sub-stocks (1, 2, 3 and 4) [12,13,33,34] (figure 1). It is likely that the individuals sampled and discussed in this paper are representatives from the sub-stocks BSB1 (Gabon) and BSC3 (Madagascar), due to the data collection locations. As there is still a degree of uncertainty regarding the geographical boundaries and the degree of interchange between the African sub-populations [13], the population-level demarcation will be used here (BSB and BSC) and discussed according to the location at which acoustic data were recorded (Gabon and Madagascar, respectively).

Song exchange between populations located in different ocean basins in the Southern Hemisphere has been documented between west and east Australian populations over multiple years [35], and between west and east African populations within a single year (2003), suggesting some degree of population connectivity [36]. Some degree of contemporary interchange has been documented previously between Gabon and Madagascar populations based on genetic studies [12–14] and direct movement based on genotypic capture–recapture of an individual whale [37]. Individual movements within a breeding season are more likely to occur between the sub-populations BSB2 (southwest coast of South Africa and Namibia) and BSC1 (southeast coast of South Africa and Mozambique) due to their relatively closer breeding ground proximity (figure 1). However, our study sites correspond with BSB1 (Gabon) and BSC3 (Madagascar) sub-stocks (figure 1), which are thought to breed at lower latitudes. Given that the African continent presents a geographical barrier between these two lower latitude breeding grounds (relative to BSB2 and BSC1), it is unlikely that an individual would visit both sites within a breeding season. Therefore, we assume that the song exchange between males in the BSB2 and BSC3 sub-stocks occurs on their shared high latitude feeding grounds, on shared migration routes and/or potentially through an individual switching breeding grounds between seasons [28,38].

Here, we investigated the degree of song sharing between the Gabon and Madagascar populations across five concurrent years (2001–2005) to investigate: (i) whether song sharing occurs between the Gabon and Madagascar populations, which have a relatively high level of genetic mixing (12–14), (ii) the degree to which songs are shared and how this varies over time, and (iii) whether measures of song similarity can be used to relate song sharing with population connectivity patterns, as observed in other Southern Hemisphere populations. This is the first study to our knowledge that uses multiple concurrent years of song data from humpback whales recorded off Africa to investigate population connectivity on contemporary time scales.

## 2. Material and methods

### 2.1. Data collection

Songs were recorded in Antongil Bay (16°00′ S, 49°55′ E), Madagascar and at two sites in Gabon: Iguela (1°51′ S, 9°20′ E) and Mayumba (3°26′ S, 10°39′ E) (figure 1). Antongil Bay is a large shallow bay in northeastern Madagascar and has a mean depth of 41.4 m [39]. Iguela and Mayumba Bays are located on the central and southern coasts of Gabon, respectively, with depths never exceeding 100 m [36].

Humpback whale songs were recorded off Madagascar using a hand-held hydrophone suspended from a 7-m fiberglass boat. The songs recorded from Gabon were obtained using a hand-held hydrophone suspended approximately 5–10 m below the surface from a 9-m rigid hull inflatable boat. In both locations, hydrophones were attached to preamplifiers and connected to a Sony TCD-D100 digital audiotape (DAT) recorder. Songs were recorded at 44.1 kHz sampling rate and 16-bit resolution [30]. The system response was flat (±3 dB) from 50 to 17 000 Hz and the overall response was as low as 20 Hz, which adequately accounts for the full range of sounds produced in humpback whale song [40]. From 2001 to 2004, DAT recordings were converted to digital wav files using Avisoft-SASLab Pro (http://www.avisoft.de/) [36]*.* Singers were not sighted and were therefore neither photographically or genetically identified during recording; each recording, or acoustic sample, represents a ‘snapshot’ of what themes were being sung during the sampling period at each location from an unknown number of singers (each acoustic sample was presumed to be from a different ‘individual singer’).

### 2.2. Acoustic analysis

Humpback whale songs are highly stereotyped [16] and are arranged in a nested hierarchy (unit, phrase, theme) [16,18,41,42]. A unit is the most fundamental level representing an individual sound. Units occur in stereotyped sequences called phrases, which can be composed of sub-phrases of one or more similar or different units which are often repeated in a sequence [21,42]. Phrases are then repeated any number of times to create themes, and themes are typically sung in a particular order to compose the song [16,18,20,21,42]. A song is defined as a stereotyped sequence of themes allowing for occasional repetition or alternations of themes within the sequence [18]*.*

### 2.3. Unit classification

**Figure 2.**
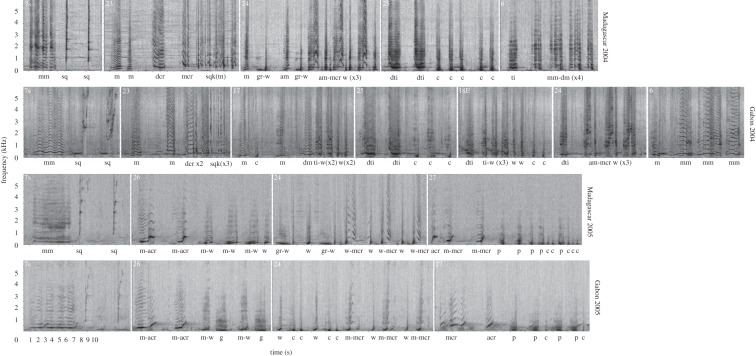
Spectrograms presenting an example of theme composition and progression of song similarity for Gabon and Madagascar songs in 2004 and 2005. In 2004, both populations shared themes 6, 7s (shifting theme), 23, 24 and 25. Themes 17 and 18E (theme evolution) were also present in Gabon 2004. In 2005, the degree of song similarity was even greater with both populations singing the same themes: 7s, 24, 26 and 27. In both years, some variability was observed in units sung between shared themes. Note that unit labels are provided below each individual unit (unit abbreviations listed in electronic supplementary material, table S1) and may differ from the median strings presented in table 2.

Songs were viewed as spectrograms in Raven Pro v. 1.5 (Hamming, 75% overlap, FFT size 1024 samples) [43]*.* Songs were transcribed manually based on the visual and acoustic qualities of the sound by a human classifier (G.A.C.) and then reviewed by a second classifier (M.L.R.). Each unit (an individual sound separated from other units by a silent period) was given a descriptive name (i.e. moan, croak, cry) based on previous descriptions from humpback whale song studies [16,18,19,21,28,30] (figure 2; electronic supplementary material, table S1). To ensure the qualitative unit-level classification was robust and repeatable within and across populations and years, 13 acoustic parameters were measured for all units sung in the first instance of each phrase type during the first song cycle recorded. For themes 1, 5, 15, 18E, 19s and 27, two examples of each phrase were measured as the units present could be more variable and this was to ensure that all unit types were measured. Time and frequency parameters of the units were measured using Raven Pro v. 1.5 (Hamming, 75% overlap, FFT size 1024 samples). The parameters measured were minimum, maximum, start and end frequencies of the units, bandwidth (maximum/minimum frequency), frequency range (ratio of minimum/maximum frequency), frequency trend (ration of start/end frequency), peak frequency (the frequency at which the maximum power occurred in the call), duration of the call(s) and the number of inflections (the change from ascending to descending frequency, or vice versa). In total, 1571 song units were measured (756 from Gabon and 815 from Madagascar). Measurements were analysed using classification and regression trees (CART) in R using the *rpart* package [44] following previous analyses [15,45,46]. *Rpart* allows statistical classification of humpback whale social calls and song units using non-parametric classification tree analysis with cross-validation [46]. CART analyses are robust to outliers, non-normal and non-independent (correlated) data, and consider all measured variables when classifying units [45–49]. The results of the CART analysis corroborated the qualitative unit-type classification (root node error: 1401/1570 = 89.23% agreement), with the majority of units grouping into the same unit types (categories) as the manual classification.

### 2.4. Classifying song similarity

Sequences of units were initially subjectively, and then quantitatively (see below), grouped into phrase types based on the sequence of units; phrases that were similar (i.e. acoustically similar units in a similar position) were grouped into themes, and themes were then grouped into songs. Phrases that changed subtly and progressively within a theme were defined as **‘**shifting themes’ [18]. Themes compared between individuals were considered to match when the patterns of units within a phrase were the same and the units themselves were very similar, allowing for a small amount of variability due to individual discrepancies [50]*.* Note that this initial qualitative grouping of phrases was used as a guide, and quantitative analyses (see below) were used to assign each sequence of units to a phrase type. Theme sequences were assigned for all full song cycles that an individual singer produced (see electronic supplementary material for all song sequences used). ‘Matching’ themes were assigned the same number and letter across singers, years and populations, and new themes were assigned a new number. Our sample size was small (table 1), particularly in some years. We have endeavoured to include as much song data as possible with the restraint that all units had to be recognizable with a good signal-to-noise ratio in a phrase to ensure a clear sequence of units (table 1).

**Table 1.** Sample sizes included for the CART and Levenshtein similarity index/Dice's similarity index analysis, and the length of recording and number of song cycles analysed per individual singer (note: individual singer numbers are arbitrary; e.g. individual 1 in 2001 Gabon is not the same individual 1 in 2003 Gabon).

	year	individual	length of recording	no. of song cycles
Gabon (BSB)	2001	1	2 h 2 min	7
		2	1 h 2 min	8
	2002	1	1 h 15 min	8
	2003	1	30 min	5
		3	22 min	1
		4	48 min	4
		6	51 min	3
	2004	1	26 min	1
		3	24 min	1
		5	52 min	3
	2005	2	1 h 31 min	5
		3	1 h 58 min	5
		7	21 min	3
		8	24 min	1
Madagascar (BSC)	2001	2	1 h 38 min	15
		3	56 min	16
	2002	1	2 h 1 min	7
		2	2 h 1 min	6
		8	50 min	3
	2003	6	1 h	2
		9	40 min	5
		10	46 min	7
		13	24 min	2
	2004	1	1 h 53 min	6
		8	1 h 49 min	5
		9	2 h 7 min	7
		11	1 h 37 min	6
	2005	10	48 min	4
		15	1 h 5 min	5
		18	2 h 1 min	6
		19	2 h 30 min	6

To validate all phrase and theme groupings, and to calculate song similarity among songs, within/between populations and across years, we used the Levenshtein distance similarity index (LSI). The LSI measures the similarity between any two sequences or strings of data, by calculating the minimum number of changes (insertions, deletions or substitutions) to change one string into the other while taking into account string length [45,51]. This analysis allows multiple strings to be compared to gain an overall understanding of sequences as well as relative levels of song similarity/difference within the dataset (see [45,52] for a detailed explanation of the calculation of the metric). Analyses were run using the custom-written software in R (see [52] for access). To ensure the qualitative classification of phrases and themes was consistent, an initial LSI analysis was run to group strings of units into phrase types (themes). This was run as a weighted analysis (*β* = 1) following [52], where unit substitution costs were based on the acoustic feature similarity of unit types (quantified as part of CART above). This removes the subjective judgement of what constitutes a ‘similar unit in a similar position’. All other operations (additions and deletions) remained as cost = 1 per change.

Once phrase types and themes were verified, song similarity among and within individual singers was investigated. We conducted two different analyses to assess song similarity. The first analysis was an unweighted LSI to compare the similarity of each singer's songs. Here, all theme sequences making up a song for each singer were compared, and the average similarity among (and within) all singers was computed (see [52]). This takes into account the variability in the theme sequences including alternations and replicates of some themes that occur in humpback song. A measure of self-similarity (i.e. how similar all songs an individual sings are) is present on the diagonal of the resultant LSI theme similarity matrix (see electronic supplementary material, table S3).

The second song similarity analysis conducted was Dice's similarity index. This analysis considered the presence (all phrase types sung by an individual singer) and sharing of phrase types in songs without including any sequential information from the songs (phrase order within song strings). Dice's similarity index (i.e. Dice's coincidence index [53]) was calculated following [15], as a measure of phrase sharing:

$$\mathrm{SI}=\frac{2A}{B+C},$$

where SI is the similarity in song phrases between individuals, *A* is the number of shared phrase types, *B* is the total number of phrase types sung by individual 1 and *C* is the total number of phrase types sung by individual 2. Dice's similarity index (Dice's SI) was calculated using the custom-written code (available at https://github.com/ellengarland/dice_si.git) and a matrix of phrase-type similarity produced.

Both the LSI theme similarity matrix and Dice's SI matrix were clustered using the *pvclust* [54] and *hclust* packages in R [52]. Dendrograms were produced using average-linkage (UPGMA, unweighted pair group method with arithmetic mean) clustering [45]. Each matrix was also bootstrapped 1000 times to assess uncertainty and the stability of the resulting structure [45]. Bootstrapping was done using both multiscale resampling (AU; significant if *p* > 95%) and normal probability (BP; significant if *p* > 70%) [15]; high AU and BP values strongly support the divisions in the tree [52]*.* Each major branch of the tree represents a song ‘type’ regardless of the population, year or individual the song came from. As an independent test of how well the tree represented the data, the cophenetic correlation coefficient (CCC) was calculated. A CCC score of 0.8 and above is considered high, and thus, a good representation of the associations present within the data [55].

## 3. Results

### 3.1. Song types

A total of 31 phrase types grouped into 27 themes were identified across all populations and years (*n* = 32 singers, table 2). Table 2 shows the most representative sequence of units making up each phrase type per population and year. One song lineage appeared to be present across all years and populations; the song evolved through the progressive addition and deletion of phrase types and themes.

**Table 2.** Median sequence of units making up each phrase type/theme per population and year. Note: theme labels = 1, 2, 3; S, shifting themes. Unit abbreviation details are provided in electronic supplementary material.

	Gabon (BSB)		Madagascar (BSC)	
year	theme	sequence	theme	sequence
2001	1	y(tn), am, dcr-w, am, dcr-w	1	gt, sn(tn), am, dcr, t, am, dcr, w, am, dcr, t
	4	w, sq, y, y, w, sq, sq, w, sq, y, y, w, sq, y, sq, y, w, sq, y, sq, y, sq, y, sq, sq	4	w, sq, y, sq, y, w, sq, y, sq, y, w, sq, y, sq, y, sq, sq, w
	5	mti-sq, sq, mti-sq, sq, mti-sq, sq, mti-sq, mti-sq, mti-sq	5	mti-sq, mti-sq, mti-sq, mti-sq, mti-sq, mti-sq, mti-sq
	11	mm, dcr, dcr-w, dcr-w	11	mm, dcr, w, dcr, w, dcr
	2	gt, gt, am, dcr-t, am, dcr-t, am, dcr-t	2	gt, gt, am, dcr, t, am, dcr, t, am, dcr, t
	3	gt, gt, mm, dcr, mm, dcr	3	gt, gt, mm, dcr, mm, dcr, mm, dcr
2002	7	pul-mm, sq, m, sq	7	mm, sq, am, sq
	7c	m, m, sq	7c	am, am, sq, sq
	8	g, am, am, sq	29	m, ti-cr, acr, dcr, dcr
	9s	m-w, ati, acr, acr, w, w, sq	12	g, ati, w, w, w
	4	w, mti-sq, sq	13	am, am, w, w, w
	10	mti-mcr, dcr, dcr	14	dcr, dcr, dcr, dcr
	14	ti, sq-m, sq-m	10	ati-mcr, dcr, dcr, dcr
	6	ti, m, m		
2003	7s	m, sq, sq	7s	mm, sq, sq
	16	m, c, m, am, ti-w	21	m, c, m, am, m-sq, m-sq
	15	m, c, m, am, m-w, m-w	16	m, acr, acr
	17	m, c, m, ti, am, m-w, m-w	15	m, w, c, m, w, am, m-w, m-w, m-w, m-w
	18	ti-w, w, w, w, c, c	14	ti, m, sq-m, sq-m
	19s	ti-w, asq, asq, asq, dsq	6	ti, m, m, m
	14	ti, sq-m, sq-m, sq-m		
	6	ti, mm, mm, mm		
	20	pul-m, pul-m, pul-m, sq, m, sq		
2004	7s	mm, sq, sq	7s	mm, sq, sq
	23	m, m, dcr, dcr, sqk, sqk, sqk	23	m, m, dcr, mcr, sqk(tn)
	17	m, c, m, dm, ti-w, ti-w, ti-w, w	24	m, gr-w, am, gr-w, am-mcr, w, am-mcr, w, am-mcr, w
	25	dti, dti, c, c, c, c	25	dti, dti, c, c, c, c
	18E	ti, ti-w, w, ti-w, w, ti-w, w, w, c, c	6	ti, m-dm, m-dm, m-dm, m-dm
	24	m, am-mcr, w, am-mcr, w, am-mcr, w, am-mcr		
	6	m, mm, mm, mm		
	20	mm, sq, m, sq		
2005	7s	mm, sq, sq	7s	mm, sq, sq
	23E	m, m, dcr-am, dcr-am, sqk(tn)	23E	m, m, m-acr, sqk, mcr, sqk(tn)
	18E	dcr-am, ti-w, w, ti-w, w, ti-w, w, ti-w, w	26	m-acr, m-acr, m-w, m-w, m-w, m-w
	26	m-acr, m-acr, m-w, gt, m-w, gt	24	w, gr-w, w, gr-w, w-mcr, w, w-mcr, w, w-mcr
	24	w, c, c, w, c, m-mcr, w, m-mcr, w, m-mcr, w	27	cr, m-acr, m-acr, p, p, p, p, p, p
	6	am, mm, mm, mm		
	27	mcr, acr, p, p, p, p, p, p		

In 2001, Gabon and Madagascar populations sang similar song types, with shared phrases/themes. This song type had five themes shared between the two populations. Two notable differences were noted between theme 1 sung in each population. First, the Gabon population sang a descending cry–woop as one unit, while the Madagascar population broke the unit into two, singing a descending cry and then a trumpet or a woop (table 2). Second, the Gabon population sang a yap train, while the Madagascar population sang a snort train after a single grunt, at the start of the theme.

In 2002, the Gabon and Madagascar populations sang varied song types, with some shared and some unique themes. Gabon's song type had eight themes, while Madagascar's song type had seven themes. Song types of both populations included a shifting theme and two phrase types for theme 7, as well as themes 10 and 14. Although these themes were present in both populations, there were variations present in units sung. For example, in theme 10, the Gabon population sang a trill-modulated cry followed by two descending cries, while the Madagascar population sang an ascending trill-modulated cry followed by three descending cries.

Gabon's 2003 song type was elaborate; it consisted of two shifting themes (7s and 19s) and seven standard themes (table 2). In comparison, the Madagascar population sang one shifting theme (7s) and five standard themes. In addition to theme 7s, Madagascar and Gabon shared themes 6, 14 and 16. The following year themes 14 and 16 were not sung in either population, while theme 6 was present in both populations' 2004 song and Gabon's 2005 song.

In 2004, all themes present in Madagascar's song type were also present in Gabon's song type, with three additional themes sung in Gabon (see tables 2 and 3, and figure 2 for further details). The shared themes were 6, 7s, 23, 24 and 25. Similar to previous years, some variability existed in the units sung per theme. For example, Gabon's theme 23 usually consisted of a moan, a descending cry, a squeak train, while in Madagascar's theme 23 the fourth unit sung was usually a modulated cry. Also, it is interesting to note that Madagascar's squeak trains were consistently longer than Gabon's squeak trains. Gabon's song type also included an evolution of theme 18 (18E), as well as themes 17 and 20.

**Table 3.** Median theme sequence of themes making up a song for each individual singer. The singer column denotes individual singer identification. Phrase repeats have been removed from the sequence.

	Gabon (BSB)		Madagascar (BSC)	
year	singer	theme sequence	singer	theme sequence
2001	1	1, 4, 5, 11, 1, 3	3	1, 2, 3, 2
	2	1, 2, 3, 2	2	1, 3, 2
2002	1	7c, 8, 10, 14, 7	1	7c, 12, 13
			2	7c, 13, 12, 13
			8	7c, 29, 12, 14, 10
2003	1	7s, 16, 18, 19s, 14, 6, 20	6	7s, 21, 15, 14
	3	7s, 16, 18, 19s, 14, 6, 20	9	7s, 21, 15, 6, 14
	4	7s, 16, 18, 19s, 14, 6, 20	10	7s, 21, 15, 14
	6	7s, 16, 15, 18, 19s, 14, 6, 20	13	7s, 21, 15, 6
2004	1	7s, 23, 17, 25, 18E, 24, 6	1	7s, 23, 24, 25, 6
	3	7s, 23, 17, 25, 24	8	7s, 23, 24, 25, 6
	5	7s, 23, 25, 18E, 24, 6	9	7s, 23, 24, 25, 6
			11	7s, 23, 24, 25, 6
2005	7	7s, 23E, 24, 6	15	7s, 26, 24, 27
	8	7s, 26, 24, 27	10	7s, 26, 24, 27
	2	7s, 26, 24	18	7s, 23E, 26, 24, 27
	3	7s, 26, 27, 24	19	7s, 26, 24, 27

Finally, in 2005, the song types from Gabon and Madagascar were overall similar (see tables 2 and 3, and figure 2 for further details). Both populations predominately sang themes 7s, 24, 26 and 27, with a few individual differences. Specifically, individual seven from Gabon sang two themes from the previous year (6 and 18E), as well as an evolution of theme 23 (23E). One individual from Madagascar (individual 18) also sang theme 23E. In general, individuals in both populations sang similar themes in the same order, with the exception of the two individuals mentioned above.

### 3.2. Song similarity

Overall, song similarity varied between individuals and therefore potentially between populations and across the years of the study. In 2001, for example, there was between 29% and 57% (LSI; Dice: 91–100%) song similarity between Gabon and Madagascar singers, whereas in 2002 there was only 21–30% song similarity (LSI; Dice: 50–62%; electronic supplementary material, table S2). However, the Gabon 2002 data contained a single singer, warranting caution with interpretation. Song similarity then progressively increased through the next 3 years between the populations (2003: 21–35% and 36–62%; 2004: 49–60% and 83–91%; 2005: 44–100% and 44–100%; LSI and Dice's SI, respectively; electronic supplementary material, table S2).

**Figure 3.**
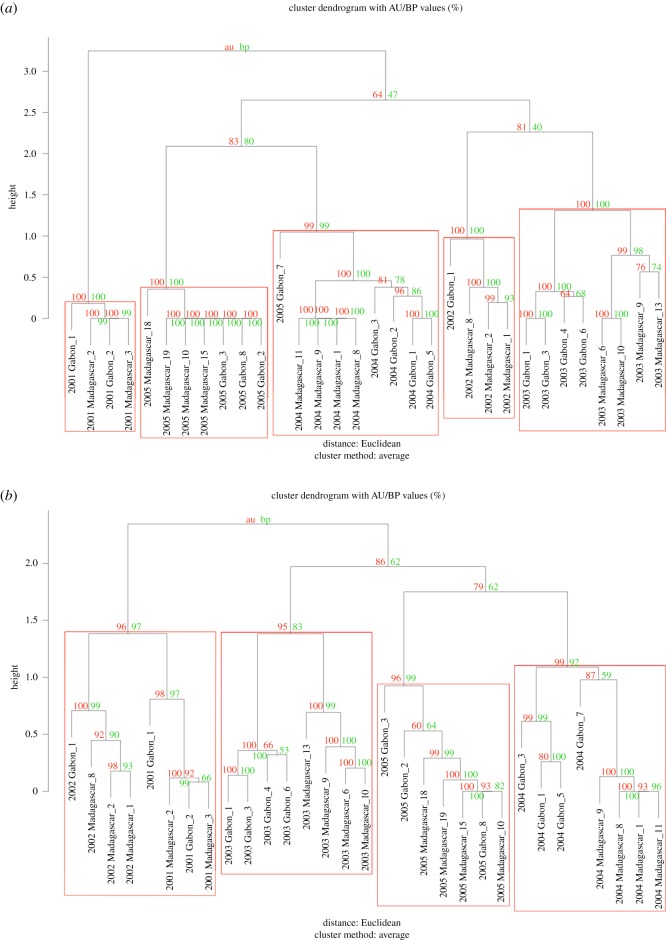
Average-linkage cluster dendrogram of the median or most representative song (theme sequence) per individual for (*a*) the Levenshtein similarity index and (*b*) Dice's similarity index. Multiscale bootstrap resampling (AU, left, red) and normal bootstrap probabilities (BP, right, green) are considered significant if *p* > 95% and if *p* > 70%, respectively. Branches with high AU values are strongly supported by the data. Individuals within a red box represent a song type and potentially a ‘vocal population’, labelled by year, location and individual number (note: individual numbers are arbitrary; e.g. individual 1 in 2001 Gabon is not the same individual in 2003 Gabon).

Clustering of the similarity matrices generated four (LSI) and five (Dice) statistically significant clusters from 5 years of song data (figure 3*a,b*), additionally confirmed using the CCC (LSI CCC = 0.9764, Dice CCC = 0.9780). Each major branch represented a song type based on song similarity; these, in turn, appeared to reflect the year of recording and not the population of recording. The LSI analysis grouped the 2001 and 2002 songs together on a higher level branch but split these into two, stable lower level clusters (figure 3*a*). Singers from both populations were mixed together within most clusters, indicating individuals in both populations sang highly similar songs each year (figure 3*a*,*b*). However, the 2003 song cluster appeared to indicate fine-scale population differentiation as individuals were separated onto two stable branches based on population in both analyses (figure 3*a*,*b*). Such fine-scale differentiation may have also been present in 2004, as singers were again split into two stable lower level clusters based on population. Finally, the placement of a Gabon 2005 singer in the 2004 song cluster suggests that this singer was singing the previous year's song. These results represent where each individual singer (or recording) from our small sample size was grouped based on the phrase types/themes recorded and the sequence in which they were sung. This small snapshot of singers from each population and year is suggestive but in no way conclusive as to the population-level interchange.

## 4. Discussion

Here, we have provided suggestive evidence of song sharing between the Madagascar and Gabon populations over several years. In general, the degree of song similarity between the Gabon and Madagascar populations is consistent with results from genetic studies suggesting a relatively high exchange rate between BSB and BSC populations, when compared to other populations within the southern Atlantic and southern Indian Oceans [12]. However, the year-to-year variability in song similarity found across the study period may indicate that these populations had more acoustic contact in some years than others, and thus opportunity for song learning and exchange. Song similarity between distinct breeding populations has generally been reported for populations inhabiting the same ocean basin, as presumably there are more opportunities for acoustic overlap of populations (i.e. [25,30,31,45,56]). The level of song sharing between the Madagascar and Gabon populations located in different ocean basins, and within the same year, is so far unreported for any other humpback whale population. The findings from this study contribute valuable information although with the necessary caution, given the sample size, towards understanding the dynamic nature of song exchange between different humpback whale breeding populations, and how patterns of song exchange may be used in conjunction with more traditional methods for understanding population structure and connectivity of humpback whales on contemporary time scales [15].

The patterns of song similarity and divergence found both in this study and others in the Southern Hemisphere (e.g. [28,30,35]), across multiple years, reinforce the idea of broad-scale connectivity in Southern Hemisphere populations [57,58]. However, there appears to be a higher level of within-year song similarity between Gabon and Madagascar than in other Southern Hemisphere populations (see [28,35,45]), but a larger sample size is warranted. There was no evidence of the song ‘revolutions’ reported between the east and west Australian populations [28,35] and neighbouring South Pacific populations [45]. A song revolution occurs when one population completely copies the song type of the neighbouring population from the year prior, leading to between-year song replacement rather than within-year song sharing [28,35,45]. Song revolutions have so far only been reported occurring in the aforementioned Southern Hemisphere populations and are not present in Northern Hemisphere populations despite numerous historical studies reporting various degrees of song sharing depending on proximity and opportunities for acoustic exchange [25,29,31]. We still know little about song learning and transmission in humpback whales, but it has been suggested that song revolutions may occur when there are a ‘threshold’ number of males singing the new song type, which then instigates song change within surrounding males, eventually spreading throughout the population [28]. One plausible explanation for the higher level of within-year song similarity found in the African populations is that there may be more opportunity for song learning on more closely aligned migration routes around the relatively narrow tip of the African continent, or on overlapping feeding grounds. Alternatively, individual singers may move more frequently between populations between years further facilitating song mixing [25].

Six distinct feeding grounds (termed ‘Areas’) are designated by the IWC that correspond with the seven breeding stocks (see electronic supplementary material). Gabon is associated with Area II, while Madagascar is associated with Area III [59]. A recent study by Amaral *et al.* [60] investigated genetic structure on Southern Hemisphere feeding grounds and found little genetic structure between areas, suggesting high levels of interchange and overlap of breeding stocks on the Southern Ocean feeding grounds [60]. Genetic diversity was higher in Areas II and III when compared with other areas, such as I (BSG) and V (BSE) [60]. Higher genetic diversity in Areas II and III, where Gabon and Madagascar are suggested to feed, could indicate mixing on feeding grounds, or a single feeding ground shared by both populations. When examining mtDNA (the maternal lineage), strong differentiation was shown between Area III and other feeding areas [60]. This suggests that mixing may be male mediated, which would allow for cultural transmission of song, a male breeding display, between breeding stocks. It remains unclear, however, whether males are coming into acoustic contact on shared migration routes, shared feeding grounds or by individuals moving between populations.

One potential driver for population mixing on feeding grounds is the movement to find limited or patchy food resources. Humpback whales may be making longer migrations due to their preys' temporal distribution, which is dependent on seasonal variables such as sea ice retreat [61,62]. The primary prey source for humpback whales is Antarctic krill (*Euphausia superba*), and feeding areas directly south of Gabon and Madagascar reportedly contain lower densities of krill, perhaps forcing movement to other areas to improve feeding opportunities [63]. Rekdahl [35] suggested that oceanographic conditions affecting prey distribution may be one factor leading to the episodic connectivity and song exchange between BSD and BSE (west and east Australian) populations. A recent study by Seyboth *et al.* [64] found significant correlations between southern right whale (*Eubalaena australis*) calving rates and krill density, providing another example of prey density and distribution effects on populations [64]. Prey distribution may therefore be one factor leading to population mixing and song sharing between Gabon and Madagascar. A tagging study by Trudelle *et al*. [65] found that humpback whales may use ocean currents to maximize movement on and around breeding grounds. Similar factors may be enabling or influencing different levels of connectivity on feeding grounds and on migration in different years. However, further research linking humpback whale movement with oceanographic variables and prey distributions is required.

Although there were no song revolutions found in the Madagascar or Gabon populations, there was an apparent song divergence in 2002, demonstrated by a reduction in song similarity relative to other years. However, the 2002 Gabon data contained a single singer, warranting caution with interpretation. A study by Darling & Sousa-Lima [57] identified song similarity between Gabon (BSB) and Brazil (BSA) in 2002, suggesting that these two breeding stocks, or at least individuals from these two populations, came into close proximity at some point during that year or the preceding year to enable song learning and matching to occur. It would seem possible therefore that there was song transmission between the Brazil and the Gabon populations that may have then led to rapid evolution and divergence of the Gabon song from 2001 to 2002. Potentially, there was directional song transmission from the 2002 Gabon song (influenced by Brazil) to Madagascar in 2003, sharing some, but not all, themes from Gabon's 2002 song and therefore not a complete song revolution (figure 3). Transmission of a song in an easterly direction has been documented to repeatedly occur between east and west Australia [35] and in the South Pacific populations [30]. Song similarity has also been documented between east Africa and west Australian populations in the southern Indian Ocean, although only one shared theme in a single year suggests limited song exchange [66]. A larger African song sample size and further research comparing song from all Southern Hemisphere populations may elucidate further complex patterns of song exchange and provide valuable information on fine-scale population connectivity patterns.

## 5. Conclusion

Our study continues to reinforce the use of acoustic methods to investigate humpback whale population connectivity, and we advocate the use of this method to support genetic and photo-identification studies. However, as with all methods, limitations do exist. Humpback whale song analysis is limited to males and therefore may not reflect female movement. Our results are suggestive of population-level interchange, but future studies are required with larger sample sizes to conclusively confirm this. On a boarder scale, our results further reinforce the complex interactions between Southern Hemisphere populations suggested by other work and have provided valuable initial information on connectivity patterns between the Gabon and Madagascan populations over yearly time scales throughout the study period. Future research studies should incorporate Brazilian song along with all other Southern Hemisphere populations in order to look at song exchange at a broader geographical level and to better understand the complex interactions and mechanisms that drive the cultural transmission of songs.

## Supplementary Material

Map of the Southern Ocean feeding grounds

## Supplementary Material

Unit abbreviation details

## Supplementary Material

All song strings for all individuals used in the Levenstein Similarity Index and the Dice's Similarity Index analysis

## Supplementary Material

Matrix of song similarity calculated using the The Levenstein Similarity Index and the Dice's Similarity Index analyses

## Data accessibility

Unit and theme sequence data are available in the electronic supplementary material. Audio files are available upon request from M.L.R. All reasonable requests for access will be honoured.
