# Supplementary figures and images for: Culturally transmitted song exchange between humpback whales (Megaptera novaeangliae) in the southeast Atlantic and southwest Indian Ocean basins
Source: R Soc Open Sci. 2018 Nov 28;5(11):172305. doi: 10.1098/rsos.172305 (PMC6281946; doi:10.1098/rsos.172305)

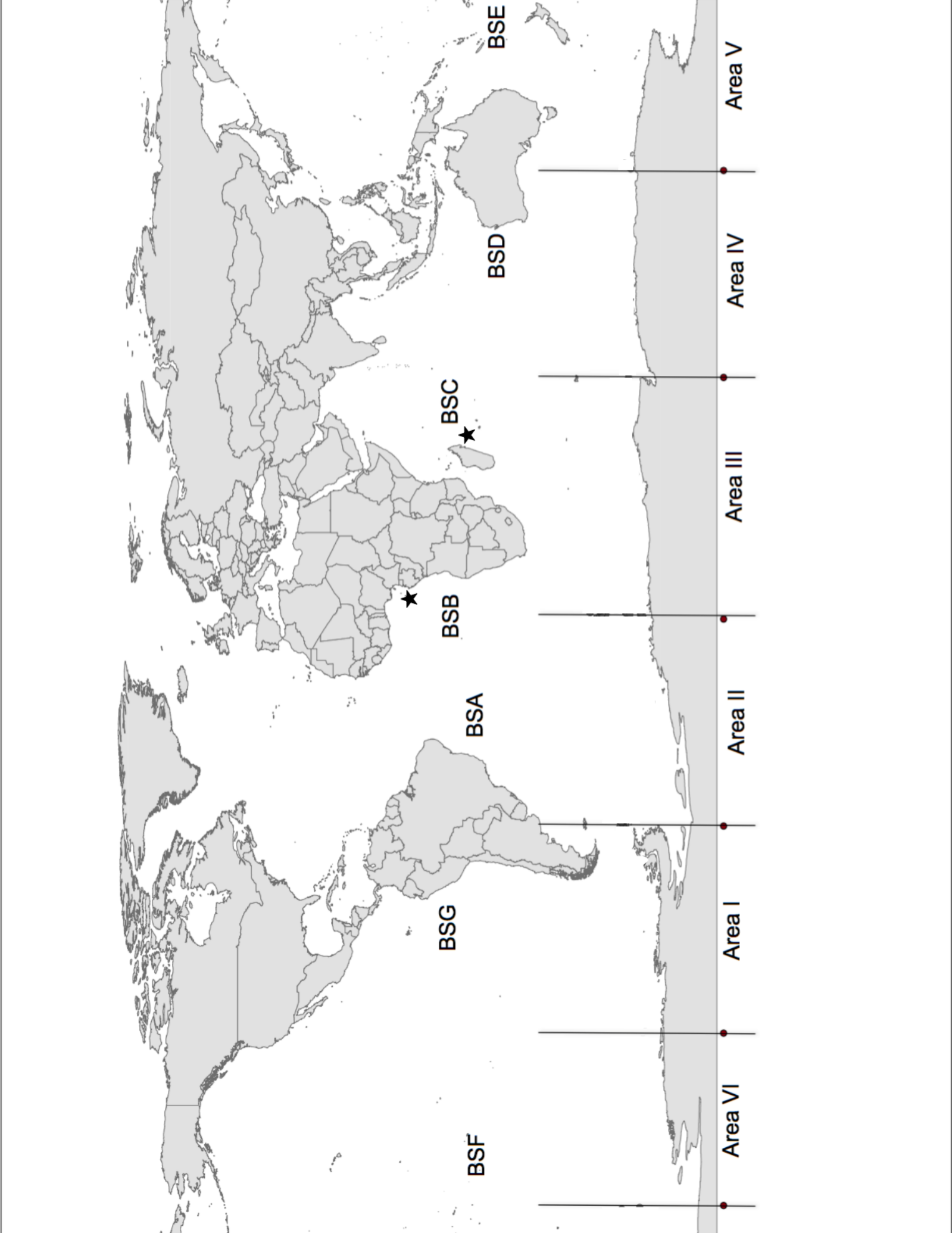

Supplement: Map of the Southern Ocean feeding grounds [file rsos172305supp1.pdf]
