# Supplementary material for: Culturally transmitted song exchange between humpback whales (Megaptera novaeangliae) in the southeast Atlantic and southwest Indian Ocean basins
Source: R Soc Open Sci. 2018 Nov 28;5(11):172305. doi: 10.1098/rsos.172305 (PMC6281946; doi:10.1098/rsos.172305)
Supplement: Unit abbreviation details [file rsos172305supp2.pdf]

Abbreviation	Sound
acr	ascending cry
am	ascending moan
am-mcr	ascending moan-modulated cry
asq	ascending squeal
ati	ascending trill
ati-mcr	ascending trill-modulated cry
c	croak
cr	cry
dcr	descending cry
dcr-am	descending cry-ascending moan
dcr-t	descending cry-trumpet
dcr-w	descending cry-woop
dm	descending moan
dsq	descending squeal
dti	descending trill
g	groan
gr	grumble
gr-w	grumble-woop
gt	grunt
m	moan
m-acr	moan-ascending cry
m-sq	moan-squeal
m-w	moan-woop
mcr	modulated cry
mm	modulated moan
mti	modulated trill
mti-mcr	modulated trill-modulated cry
mti-sq	modulated trill-squeal
p	purr
pul-mm	pulsative modulated moan
rcht	rachet
sn(tn)	snort train
sq	squeal
sq-m	squeal-moan
sqk	squeak
sqk(tn)	squeak train
t	trumpet
ti	trill
ti-cr	trill-cry
ti-mcr	trill-modulated cry
ti-w	trill-woop
w	woop
w-mcr	woop-modulated cry
y	yap
y(tn)	yap train
