# Supplementary material for: Culturally transmitted song exchange between humpback whales (Megaptera novaeangliae) in the southeast Atlantic and southwest Indian Ocean basins
Source: R Soc Open Sci. 2018 Nov 28;5(11):172305. doi: 10.1098/rsos.172305 (PMC6281946; doi:10.1098/rsos.172305)
Supplement: All song strings for all individuals used in the Levenstein Similarity Index and the Dice's Similarity Index analysis [file rsos172305supp3.pdf]

	Year	Individual	Song #	Theme sequence
Gabon (BSB)	**2001**	1	1	1, 4, 5, 11, 1, 3
			2	1, 4, 5, 11, 1, 3
			3	1, 4, 5, 11, 1, 3
			4	1, 4, 5, 1, 3
			5	1, 4, 5, 1, 3
			6	1, 4, 5, 11, 1, 3
			7	1, 4, 5, 11, 1, 3
		2	1	1, 2, 3, 2
			2	1, 2, 3, 2
			3	1, 4, 5, 11
			4	1, 2, 3, 2
			5	1, 2
			6	1, 3, 2
			7	1, 2, 4, 5, 11
			8	1, 3, 2
	**2002**	1	1	7c, 8, 10, 14, 6
			2	7c, 8, 14, 7
			3	7c, 8, 9s, 10, 14, 6, 7
			4	7c, 8, 10, 14, 7
			5	7c, 8, 9s, 4, 10, 14, 7
			6	7c, 8, 9s, 14, 6, 7
			7	7c, 8, 9s, 4, 10, 14
	**2003**	1	1	7s, 16, 18, 19s, 6, 20
			2	7s, 16, 18, 19s, 14, 6, 20
			3	7s, 16, 18, 19s, 14, 6, 20
			4	7s, 16, 18, 19s, 14, 6, 20
			5	7s, 16, 18, 19s, 6, 20
		3	1	7s, 16, 18, 19s, 14, 6, 20
		4	1	7s, 16, 17, 18, 19s, 14, 6, 20
			2	7s, 16, 18, 19s, 14, 6, 20
			3	7s, 16, 15, 18, 19s, 14, 6
			4	18, 19s, 14, 6, 20
		6	1	7s, 16, 15, 18, 19s, 14, 6, 20
			2	7s, 16, 15, 18, 19s, 14, 6, 20
			3	7s, 16, 15, 18, 19s, 14, 6
	**2004**	1	1	7s, 23, 17, 25, 18E, 24, 6
		3	1	7s, 23, 17, 25, 24
		5	1	7s, 23, 17, 25, 18E, 24, 6
			2	7s, 23, 25, 18E, 24
			3	7s, 23, 25, 18E, 24, 6
	**2005**	2	1	7s, 26, 24, 27

			2	7s, 26, 24, 26, 24, 27
			3	7s, 26, 24
			4	7s, 26, 24, 26, 24
			5	7s, 26, 24
		3	1	7s, 26, 27, 24
			2	7s, 26, 27, 24
			3	7s, 26, 24, 27, 26, 24
			4	7s, 26, 27, 24
			5	7s, 26, 27, 24, 27, 24
		7	1	7s, 23E, 24, 18E, 24, 6
			2	7s, 23E, 24, 6
			3	7s, 23E, 24, 6
		8	1	7s, 26, 24, 27
**Madagascar (BSC)**	**2001**	2	1	1, 2
			2	1, 4, 5, 11
			3	1, 3, 2, 3, 2, 3, 2, 4, 5, 11
			4	1, 3, 2
			5	1, 2, 3, 2
			6	1, 3, 2, 3
			7	1, 3
			8	1, 4, 5, 11, 3, 2
			3	1, 3, 2
			10	1, 3, 2
			11	1, 3, 2
			12	1, 4, 5, 11
			13	1, 3, 2
			14	1, 3, 2
			15	1, 3
		3	1	1, 2, 3, 2
			2	1, 2, 3, 2
			3	1, 2, 3
			4	1, 2, 3, 2
			5	1, 4, 5, 11
			6	1, 2, 3, 2
			7	1, 2, 3
			8	1, 2, 3, 2
			3	1, 2, 3, 2
			10	1, 2, 3
			11	1, 3, 2
			12	1, 3, 2, 4, 5, 11
			13	2, 3, 2, 3
			14	1, 2, 3, 2
			15	3, 2, 3

			16	1, 4, 5, 11, 2, 3
	2002	1	1	7c, 12, 13
			2	7c, 14, 7,
			3	7c, 12, 13
			4	7c, 13, 12
			5	7c, 14, 10
			6	7c, 13
			7	7c, 14, 10
		2	1	7c, 13, 12, 13
			2	7c, 12, 13, 12
			3	7c, 12
			4	7c, 13, 12, 13
			5	7c, 13, 12, 13, 12, 13
			6	7c, 13, 14, 10
		8	1	7c, 29, 14, 10
			2	7c, 29, 12, 14,10
			3	7c, 29, 12, 14, 10
	2003	6	1	7s, 21, 15, 14
			2	7s, 15, 14
		9	1	7s, 21, 15, 6, 14
			2	7s, 21, 15, 14, 6
			3	7s, 21, 15, 6, 14
			4	7s, 21, 15, 6, 14
			5	7s, 21, 15, 6, 14, 6
		10	1	7s, 21, 15, 14
			2	7s, 21, 15, 14, 15, 14
			3	7s, 21, 15, 14
			4	7s, 21, 15
			5	7s, 21, 15, 14
			6	7s, 21, 15, 14
			7	7s, 21, 15
		13	1	7s, 21, 15, 6
			2	7s, 6
	2004	1	1	7s, 23, 24, 25, 6
			2	7s, 23, 24, 25, 6
			3	7s, 23, 24, 25, 6
			4	7s, 23, 24, 25, 6
			5	7s, 23, 24, 25, 6
			6	7s, 23, 24, 25, 6
		8	1	7s, 23, 24, 25, 6
			2	7s, 23, 24, 25, 6
			3	7s, 23, 24, 25, 6
			4	7s, 23, 24, 25, 6

			5	7s, 23, 24, 25, 6
		9	1	7s, 23, 24, 25, 6
			2	7s, 23, 24, 25, 6
			3	7s, 23, 24, 25, 6
			4	7s, 23, 24, 25, 6
			5	7s, 23, 24, 25, 6
			6	7s, 23, 24
			7	7s, 23, 24, 25, 6
		11	1	7s, 23, 24, 25, 6
			2	7s, 23, 24, 25, 6
			3	7s, 23, 24, 25, 6
			4	7s, 23, 24, 25, 6
			5	7s, 23, 24, 25, 6
			6	7s, 23, 24, 25, 6
	**2005**	10	1	7s, 26, 24, 27
			2	7s, 26, 24, 27
			3	7s, 26, 24, 27
			4	7s, 26, 24, 27
		15	1	7s, 26, 24, 27
			2	7s, 26, 24, 27
			3	7s, 26, 24, 27
			4	7s, 26, 24, 27
			5	7s, 26, 24, 27
		18	1	7s, 23E, 26, 24, 27
			2	7s, 26, 24, 27
			3	7s, 23E, 26, 24, 27
			4	7s, 26, 24, 27
			5	7s, 26, 24, 27
			6	7s, 23E, 26, 24, 27
		19	1	7s, 26, 24, 27
			2	7s, 26, 24, 27
			3	7s, 26, 24, 27
			4	7s, 24, 27
			5	7s, 26, 24, 27
			6	7s, 26, 24, 27
