# Supplementary material for: Culturally transmitted song exchange between humpback whales (Megaptera novaeangliae) in the southeast Atlantic and southwest Indian Ocean basins
Source: R Soc Open Sci. 2018 Nov 28;5(11):172305. doi: 10.1098/rsos.172305 (PMC6281946; doi:10.1098/rsos.172305)
Supplement: Matrix of song similarity calculated using the The Levenstein Similarity Index and the Dice's Similarity Index analyses [file rsos172305supp4.pdf]

DICE/LSI	GA.01.1	GA.01.2	MA.01.3	MA.01.2	GA.02.1	MA.02.2	MA.02.1	MA.02.8	GA.03.1	GA.03.3	GA.03.4	GA.03.6	MA.03.6	MA.03.9	MA.03.10	MA.03.13	GA.04.1	GA.04.2	GA.04.3	GA.04.5	MA.04.1	MA.04.8	MA.04.9	MA.04.11	GA.05.7	GA.05.8	GA.05.2	GA.05.3	MA.05.15	MA.05.10	MA.05.18	MA.05.19
GA.01.1	**1/0.93**	0.27	0.29	0.31	0.01	0	0	0	0	0	0	0	0	0	0	0	0	NA	0	0	0	0	0	0	0	0	0	0	0	0	0	0
GA.01.2	0.91	**1/0.61**	0.57	0.56	0.01	0	0	0	0	0	0	0	0	0	0	0	0	NA	0	0	0	0	0	0	0	0	0	0	0	0	0	0
MA.01.3	0.91	1	**1/0.62**	0.51	0.01	0	0	0	0	0	0	0	0	0	0	0	0	NA	0	0	0	0	0	0	0	0	0	0	0	0	0	0
MA.01.2	0.91	1	1	**1/0.59**	0.01	0	0	0	0	0	0	0	0	0	0	0	0	NA	0	0	0	0	0	0	0	0	0	0	0	0	0	0
GA.02.1	0	0	0	0	**1/0.69**	0.21	0.27	0.30	0.13	0.16	0.16	0.17	0.16	0.13	0.11	0.07	0.02	NA	0	0.02	0.07	0.07	0.06	0.07	0.06	0	0	0	0	0	0	0
MA.02.2	0	0	0	0	0.50	**1/0.59**	0.47	0.38	0.01	0.02	0.03	0.02	0.02	0.01	0	0	0	NA	0	0	0	0	0	0	0	0	0	0	0	0	0	0
MA.02.1	0	0	0	0	0.62	0.91	**1/0.55**	0.40	0.04	0.06	0.07	0.06	0.00	0.03	0.01	0	0	NA	0	0	0	0	0	0	0	0	0	0	0	0	0	0
MA.02.8	0	0	0	0	0.50	0.80	0.73	**1/0.76**	0.06	0.10	0.09	0.09	0.11	0.05	0.05	0	0	NA	0	0	0	0	0	0	0	0	0	0	0	0	0	0
GA.03.1	0	0	0	0	0.29	0.17	0.15	0.17	**1/0.93**	0.94	0.76	0.77	0.24	0.33	0.21	0.30	0.14	NA	0.15	0.18	0.30	0.30	0.28	0.30	0.28	0.15	0.15	0.15	0.15	0.15	0.15	0.15
GA.03.3	0	0	0	0	0.29	0.17	0.15	0.17	1	**1**	0.83	0.82	0.29	0.34	0.24	0.29	0.14	NA	0.14	0.19	0.29	0.29	0.27	0.29	0.29	0.14	0.14	0.14	0.14	0.14	0.14	0.14
GA.03.4	0	0	0	0	0.25	0.14	0.13	0.14	0.88	0.88	**1/0.76**	0.78	0.27	0.33	0.22	0.27	0.20	NA	0.13	0.17	0.21	0.21	0.19	0.21	0.24	0.10	0.10	0.10	0.10	0.10	0.10	0.10
GA.03.6	0	0	0	0	0.27	0.15	0.14	0.15	0.93	0.93	0.94	**1/0.94**	0.39	0.45	0.36	0.33	0.26	NA	0.13	0.22	0.26	0.26	0.24	0.26	0.26	0.13	0.13	0.13	0.13	0.13	0.13	0.13
MA.03.6	0	0	0	0	0.18	0.22	0.20	0.22	0.36	0.36	0.46	0.50	**1/0.88**	0.68	0.74	0.46	0.14	NA	0.20	0.17	0.20	0.20	0.21	0.20	0.22	0.25	0.24	0.22	0.25	0.25	0.23	0.26
MA.03.9	0	0	0	0	0.33	0.20	0.18	0.20	0.50	0.50	0.57	0.62	0.89	**1/0.85**	0.70	0.58	0.20	NA	0.19	0.21	0.27	0.27	0.26	0.27	0.34	0.19	0.19	0.18	0.19	0.19	0.19	0.19
MA.03.10	0	0	0	0	0.18	0.22	0.20	0.22	0.36	0.36	0.46	0.50	1	0.89	**1/0.82**	0.49	0.14	NA	0.20	0.17	0.20	0.20	0.20	0.20	0.21	0.24	0.22	0.21	0.24	0.24	0.22	0.24
MA.03.13	0	0	0	0	0.17	0	0	0	0.50	0.50	0.57	0.62	0.67	0.80	0.67	**1/0.75**	0.29	NA	0.20	0.27	0.40	0.40	0.38	0.40	0.44	0.25	0.24	0.22	0.25	0.25	0.23	0.26
GA.04.1	0	0	0	0	0.14	0	0	0	0.29	0.29	0.38	0.27	0.18	0.33	0.18	0.33	**1**	NA	0.71	0.86	0.57	0.57	0.55	0.57	0.48	0.29	0.29	0.29	0.29	0.29	0.29	0.29
GA.04.2	0	0	0	0	0.15	0	0	0	0.31	0.31	0.40	0.29	0.20	0.36	0.20	0.36	0.92	**1**	NA	NA	NA	NA	NA	NA	NA	NA	NA	NA	NA	NA	NA	NA
GA.04.3	0	0	0	0	0.14	0	0	0	0.43	0.43	0.50	0.40	0.18	0.33	0.18	0.33	0.86	0.92	**1**	0.60	0.60	0.60	0.60	0.60	0.24	0.20	0.35	0.37	0.20	0.20	0.20	0.20
GA.04.5	0	0	0	0	0.14	0	0	0	0.29	0.29	0.38	0.27	0.18	0.33	0.18	0.33	1	0.92	0.86	**1/0.87**	0.49	0.49	0.49	0.49	0.44	0.27	0.32	0.31	0.27	0.27	0.27	0.27
MA.04.1	0	0	0	0	0.17	0	0	0	0.33	0.33	0.29	0.31	0.22	0.40	0.22	0.40	0.83	0.91	0.83	0.83	**1**	1	0.94	1.00	0.57	0.40	0.39	0.25	0.40	0.40	0.30	0.40
MA.04.8	0	0	0	0	0.17	0	0	0	0.33	0.33	0.29	0.31	0.22	0.40	0.22	0.40	0.83	0.91	0.83	0.83	1	**1**	0.94	1.00	0.57	0.40	0.39	0.25	0.40	0.40	0.30	0.40
MA.04.9	0	0	0	0	0.17	0	0	0	0.33	0.33	0.29	0.31	0.22	0.40	0.22	0.40	0.83	0.91	0.83	0.83	1	1	**1/0.90**	0.94	0.55	0.41	0.40	0.28	0.41	0.41	0.32	0.41
MA.04.11	0	0	0	0	0.17	0	0	0	0.33	0.33	0.29	0.31	0.22	0.40	0.22	0.40	0.83	0.91	0.83	0.83	1	1	1	**1**	0.57	0.40	0.39	0.25	0.40	0.40	0.30	0.40
GA.05.7	0	0	0	0	0.17	0	0	0	0.33	0.33	0.29	0.31	0.22	0.40	0.22	0.40	0.67	0.55	0.50	0.67	0.60	0.60	0.60	0.60	**1/0.85**	0.44	0.43	0.30	0.44	0.44	0.51	0.44
GA.05.8	0	0	0	0	0	0	0	0	0.18	0.18	0.15	0.17	0.25	0.22	0.25	0.22	0.36	0.40	0.36	0.36	0.44	0.44	0.44	0.44	0.44	**1**	0.75	0.57	1	1.00	0.90	0.96
GA.05.2	0	0	0	0	0	0	0	0	0.18	0.18	0.15	0.17	0.25	0.22	0.25	0.22	0.36	0.40	0.36	0.36	0.44	0.44	0.44	0.44	0.44	1	**1/0.74**	0.61	0.75	0.75	0.68	0.71
GA.05.3	0	0	0	0	0	0	0	0	0.18	0.18	0.15	0.17	0.25	0.22	0.25	0.22	0.36	0.40	0.36	0.36	0.44	0.44	0.44	0.44	0.44	1	1	**1/0.81**	0.57	0.57	0.50	0.56
MA.05.15	0	0	0	0	0	0	0	0	0.18	0.18	0.15	0.17	0.25	0.22	0.25	0.22	0.36	0.40	0.36	0.36	0.44	0.44	0.44	0.44	0.44	1	1	1	**1**	1	0.90	0.96
MA.05.10	0	0	0	0	0	0	0	0	0.18	0.18	0.15	0.17	0.25	0.22	0.25	0.22	0.36	0.40	0.36	0.36	0.44	0.44	0.44	0.44	0.44	1	1	1	1	**1**	0.90	0.96
MA.05.18	0	0	0	0	0	0	0	0	0.17	0.17	0.14	0.15	0.22	0.20	0.22	0.20	0.33	0.36	0.33	0.33	0.40	0.40	0.40	0.40	0.60	0.89	0.89	0.89	0.89	0.89	**1/0.9**	0.86
MA.05.19	0	0	0	0	0	0	0	0	0.18	0.18	0.15	0.17	0.25	0.22	0.25	0.22	0.36	0.40	0.36	0.36	0.44	0.44	0.44	0.44	0.44	1	1	1	1	1	0.89	**1/0.93**
